# The Dementia Palliative Care Clinical Trials Training Program

**DOI:** 10.1093/geroni/igaf122.1510

**Published:** 2025-12-31

**Authors:** Zachary Baker, Emily Mroz, Jean Kutner, Kathryn Pollack, Christine Ritchie, Ana-Maria Vranceanu

**Affiliations:** Arizona State University, Phoenix, Arizona, United States; Emory University, Atlanta, Georgia, United States; University of Colorado School of Medicine, Aurora, Colorado, United States; Duke University School of Medicine, Durham, North Carolina, United States; Massachusetts General Hospital, Boston, Massachusetts, United States; Massachusetts General Hospital, Boston, Massachusetts, United States

## Abstract

The Dementia Palliative Care Clinical Trials Training Program (DEM-PCCT) is a 10-month intensive program designed to equip early- and mid-career researchers with the skills necessary to design and conduct clinical trials in the dementia palliative care space. The program integrates large cohort-wide meetings, monthly small-group mentoring, and a five-day in-person institute. Scholars receive hands-on training in trial methodology, grant writing, and research ethics while building a strong professional network. A core component is the development of a competitive funding proposal for a dementia-related palliative care clinical trial. Since its inception, 53 scholars across three cohorts have completed DEM-PCCT. Of these, a growing number have secured funding, initiated collaborations, contributed to dementia palliative care research, and increased confidence and comfort with conducting clinical trials (p<.05). This presentation highlights two concrete examples of submissions to the National Institute on Aging: (1) the first author’s R01 application, currently under review, and (2) the second author’s K22 application, which has been successfully funded. Both applications marked first-time submissions for the Principal Investigators, underscoring the program’s role in fostering emerging researchers. DEM-PCCT’s success is driven by several key elements: a) Structured mentorship providing individualized guidance, b) Access to previously funded trial materials and methods, allowing scholars to build on successful frameworks, c) Regular deadlines and peer feedback to ensure steady progress toward grant submission, and d) Statistical mentorship, a unique feature often missing from other dementia training programs. As part of a broader symposium, this talk will examine how DEM-PCCT differs from other programs.

